# Clinical Evaluation of Patients with Genetically Confirmed Familial Hypercholesterolemia

**DOI:** 10.3390/jcm12031030

**Published:** 2023-01-29

**Authors:** Andrea Aparicio, Francisco Villazón, Lorena Suárez-Gutiérrez, Juan Gómez, Ceferino Martínez-Faedo, Edelmiro Méndez-Torre, Pablo Avanzas, Rut Álvarez-Velasco, Elías Cuesta-Llavona, Claudia García-Lago, David Neuhalfen, Eliecer Coto, Rebeca Lorca

**Affiliations:** 1 Área del Corazón, Hospital Universitario Central Asturias (HUCA), 33011 Oviedo, Spain; 2 Servicio de Endocrinología y Nutrición, Hospital Universitario Central Asturias (HUCA), 33011 Oviedo, Spain; 3 Instituto de Investigación Sanitaria del Principado de Asturias (ISPA), 33011 Oviedo, Spain; 4 Departamento de Genética Molecular, Hospital Universitario Central Asturias (HUCA), 33011 Oviedo, Spain; 5 Redes de Investigación Cooperativa Orientadas a Resultados en Salud (RICORs), 28029 Madrid, Spain; 6 Unidad de Cardiopatías Familiares, Hospital Universitario Central Asturias (HUCA), 33011 Oviedo, Spain; 7 CIBER-Enfermedades Respiratorias, 28029 Madrid, Spain; 8 Medicine Department, Universidad de Oviedo, 33003 Oviedo, Spain; 9 Departamento de Morfología y Biología Celular, Universidad de Oviedo, 33003 Oviedo, Spain

**Keywords:** familial hypercholesterolemia (FH), atherosclerotic cardiovascular disease (ASCVD), genetic testing, cardiovascular prevention

## Abstract

Familial hypercholesterolemia (FH) is the most common genetic disorder associated with premature atherosclerotic cardiovascular (CV) disease (ASCVD). However, it still is severely underdiagnosed. Initiating lipid-lowering therapy (LLT) in FH patients early in life can substantially reduce their ASCVD risk. As a result, identifying FH is of the utmost importance. The increasing availability of genetic testing may be useful in this regard. We aimed to evaluate the genetic profiles, clinical characteristics, and gender differences between the first consecutive patients referred for genetic testing with FH clinical suspicion in our institution (a Spanish cohort). Clinical information was reviewed, and all participants were sequenced for the main known genes related to FH: *LDLR*, *APOB*, *PCSK9* (heterozygous FH), *LDLRAP1* (autosomal recessive FH), and two other genes related to hyperlipidaemia (APOE and *LIPA*). The genetic yield was 32%. Their highest recorded LDLc levels were 294 ± 65 SD mg. However, most patients (79%) were under > 1 LLT medication, and their last mean LDLc levels were 135 ± 51 SD. *LDLR* c.2389+4A>G was one of the most frequent pathogenic/likely pathogenic variants and its carriers had significantly worse LDLc highest recorded levels (348 ± 61 SD vs. 282 ± 60 SD mg/dL, *p* = 0.002). Moreover, we identified an homozygous carrier of the pathogenic variant *LDLRAP1* c.207delC (autosomal recessive FH). Both clinical and genetic hypercholesterolemia diagnosis was significantly established earlier in men than in women (25 years old ± 15 SD vs. 35 years old ± 19 SD, *p* = 0.02; and 43 ± 17 SD vs. 54 ± 19 SD, *p* = 0.02, respectively). Other important CV risk factors were found in 44% of the cohort. The prevalence of family history of premature ASCVD was high, whereas personal history was exceptional. Our finding reaffirms the importance of early detection of FH to initiate primary prevention strategies from a young age. Genetic testing can be very useful. As it enables familial cascade genetic testing, early prevention strategies can be extended to all available relatives at concealed high CV risk.

## 1. Introduction

According to the last European guidelines on cardiovascular disease prevention, atherosclerotic cardiovascular (CV) disease (ASCVD) is still a major cause of morbidity and mortality. Smoking cessation, the adoption of a healthy lifestyle, and risk factor treatment is recommended for all patients [1]. In addition, dyslipidaemia is known to be one of the main causal but also modifiable ASCVD risk factors [1]. Low-density lipoprotein cholesterol (LDLc), along with other apoB-containing lipoproteins, has demonstrated a causal role in the development of ASCVD [1,2]. Randomised controlled trials (RCTs) have proven that lowering LDLc levels with lipid-lowering therapy (LLT) safely reduces ASCVD risk, even at extremely low LDLc levels [1,2]. The absolute benefit of lowering LDLc depends on the absolute risk of ASCVD and the absolute reduction in LDLc [1]. In fact, even a small absolute reduction in LDLc may be beneficial in high or very high-risk patients [3].

In this regard, some patients with dyslipidaemia could have an inherited genetic disorder: familial hypercholesterolemia (FH). Initiating LLT in FH patients early in life can substantially reduce their ASCVD risk [4]. However, untreated young adults with FH could have a 90-fold increase in ASCVD mortality [5]. Therefore, it is not surprising that current European guidelines consider FH patients directly as ASCVD high-risk patients [1]. As a result, early identification of FH has a potential important impact for clinical management and public health [6]. Prevention strategies on the prompt diagnosis of FH and accurate LLT treatment are of the utmost importance.

Historically, the diagnosis of FH has been based on clinical diagnostic criteria, such as the Dutch Lipid Clinic Network Diagnostic Criteria (DLCN) [7,8] or the Simon Broome Register Diagnostic Criteria [9]. Clinical data have included information about personal or family history of premature ASCVD, personal or family elevated LDLc levels, and physical examination (including corneal arcus or tendon xanthomas), among others [10,11]. Although FH diagnosis can be made based on clinical findings alone, genetic testing can be key to achieving the definite diagnosis [10]. In fact, identifying pathogenic variants of FH in genetic testing has been considered the “gold standard” for FH diagnosis [12].

Although FH is the most common genetic disorder associated with premature ASCVD [13] and one of the most common genetic conditions, with a prevalence of 1/250–1/500 individuals [6,14,15], it still is severely underdiagnosed [16]. The increasing availability, accessibility, and quality of genetic testing worldwide may improve this issue. Accordingly, next-generation sequencing (NGS) has recently been implemented in our centre for FH diagnosis, without the need for further external referral.

In this scenario, we aimed to evaluate the genetic profiles, clinical characteristics, and gender differences between the first consecutive patients referred for genetic testing with an FH diagnosis in our institution.

## 2. Materials and Methods

### 2.1. Study Population

In this retrospective study, we reviewed all consecutive patients referred for genetic testing due to clinical suspicion of FH, from 2018 to 2022, in a Spanish national reference centre for inherited cardiac conditions.

We retrospectively collected clinical data from this cohort. We reviewed their birth data, age at first dyslipidaemia diagnosis, and the age of definite genetic diagnosis. Historically higher levels and the most recent ones of total cholesterol (TC), LDLc, high-density lipoprotein cholesterol (HDLc), and triglycerides (TGs) were evaluated. Current LLT was also reviewed. In addition, classical cardiovascular risk factors such as high blood pressure (HBP), tobacco consumption, diabetes mellitus (DM), dyslipidaemia, body mass index (BMI), renal failure, and personal or familiar ASCVD were also collected. Lipoprotein A (LPa) levels were reviewed, when available.

Clinical suspicion of FH was evaluated according to the Dutch Lipid Clinical Network (DLCN) criteria. Punctuation was obtained via direct (reflected in clinical history) or indirect calculation (calculated by the investigators based on available clinical data). Premature cardiovascular disease was considered, according to the DLCN definition, in men <55 years old and women <60 years old [1].

All patients who wished to participate had signed written consent to grant access to their genetic data for investigational purposes. The research protocol followed institutional ethics guidelines. This study was evaluated by the local Ethical Committee (CEImPA 2022.254).

### 2.2. Genetic Testing

Blood samples were obtained from all patients who accepted undergoing genetic testing, collected in a 9 mL tube with EDTA anticoagulant. DNA was isolated from their peripheral blood leukocytes via the standard salting-out method, a simple and non-toxic DNA extraction technique that isolates high-quality DNA from the total blood [17].

All genes related to FH were evaluated: *LDLR*, *APOB*, *PCSK9* (heterozygous FH), *LDLRAP1* (autosomal recessive FH), and 2 other genes related to hyperlipidaemia (APOE and *LIPA*). All patients were sequenced via NGS with a total of 210 genes that were associated with cardiovascular disease, including FH-associated genes. The genetic detailed procedure has been previously reported elsewhere [18,19,20,21]. All participants were NGS sequenced for the same gene panel, including the coding sequence plus at least 5 flanking intronic base pairs of *LDLR*, *APOB*, *PCSK9*, *APOE*, *LDLRAP1*, and *LIPA* genes via Ion Torrent semiconductor chip technology in an Ion GeneStudio S5 Sequencer (Thermo Fisher Scientific, Waltham, MA, USA), according to previously described protocols [18,19,20,21]. Overall, in silico coverage of the included genes was 100%. Variant Caller v5 software was used for variant identification (Thermo Fisher Scientific). Ion Reporter (Thermo Fisher Scientific, Waltham, MA, USA) and HD Genome One (DREAMgenics S.L., www.dreamgenics.com, Oviedo, Spain) software were used for variant annotation, including population, functional, disease-related, and in silico predictive algorithm databases.

Interpretation of all gene variants with an allele frequency of <0.01 in the gnomAD European non-Finnish database was based on the American College of Medical Genetics and Genomics (ACMG-AMP) 2015 Standards and Guidelines [22]. According to ACMG-AMP criteria, they were classified as likely pathogenic/pathogenic variants (LP/P), and variants of uncertain significance (VUS). If only benign or likely benign variants were found, they were not reported, and the genetic result was informed as negative.

**Figure 1:**
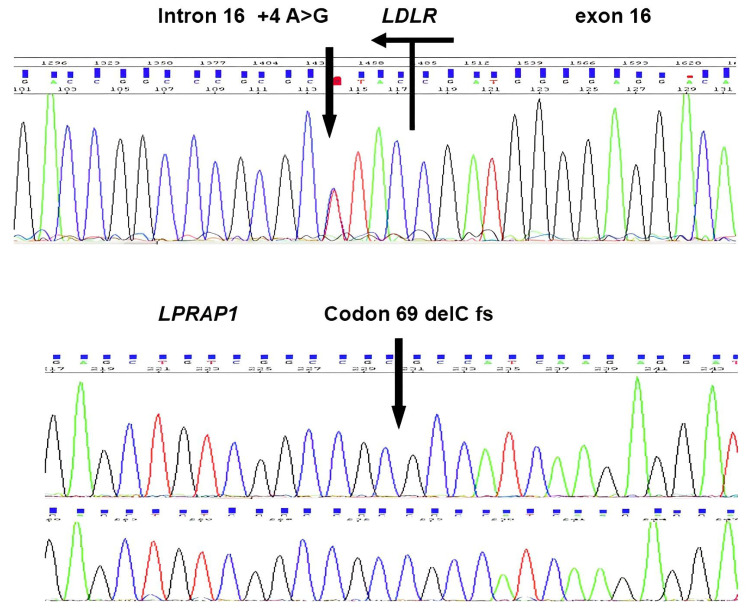
Sanger sequencing of one of the multiple carriers of *LDLR*, *c*.2389+4A>G, and the homozygous carrier of *LDLRAP1:* c.207delC variant. For the *LDLR*, we show the reverse strand sequence with the exon 16-intron 16 site.

Variants of interest classified as likely pathogenic were confirmed in the corresponding patients via capillary Sanger sequencing of PCR fragments (Figure 1).

### 2.3. LDLR Intron 16 Variant, Transcript Analysis

We hypothesised that the LDLR intron 16 + 4 (c.2389+4A>G) change might affect the pre-mRNA splicing. To confirm this, we amplified and sequenced a fragment from leukocyte cDNA. Briefly, total RNA was isolated from leukocytes in 5 mL of blood. The RNA was reverse transcribed (High-Capacity cDNA Reverse Transcription Kit, Life Technologies, Carlsbad, CA, USA) and the cDNA was amplified with primers that matched exons 14–15 and 17–18.

To support the variant pathogenicity, its prevalence was evaluated among 500 blood donors from our region, without known history of FH and LDLc values <150 mg/dL. They were genotyped using Real Time PCR with Taqman probes that recognised the wild-type (A) and mutation (G) alleles.

### 2.4. Statistical Analysis

Statistical analyses were performed using SPSS v.19. Descriptive data for continuous variables are presented as mean ± SD and as frequencies or percentages for categorical variables. The chi-square test or Fisher exact test was used to compare frequencies, whereas differences in continuous variables were evaluated with either the Student t-test or the Mann–Whitney U test. *p* < 0.05 was considered to be significant.

## 3. Results

Due to clinical suspicion of FH based on LDLc levels and clinical findings, 182 Spanish Caucasian patients were referred for genetic testing.

Genetic testing identified a genetic LP/P variant in 58 patients. As a genetic final diagnosis of FH was established in 58 patients, the genetic yield of the cohort was 32%. All genetic relevant variants are shown in Table 1. In two patients who were already carriers of a pathogenic variant in *LDLR* that could solely explain their FH phenotype, an additional VUS was found: one was a carrier of the P variant *LDLR* p.Gly592Glu and a VUS (*LDLR* Arg253Gln); and another was a carrier of the P variant *LDLR* p.Glu288Lys and a VUS in *APOB* (*APOB* Asp1908Asn). The clinical significance of these additional VUS is unknown.

In the remaining 124 patients, genetic results were either negative or inconclusive (carriers of either benign/likely benign variants or variants of unknown significance). In those patients with negative results, the historical LDLc meant that the highest levels were 242 mg/dL ± 75 SD. From them, 49% had an at-least-probable clinical diagnosis of FH according to the DLCN criteria (score of 6 or higher). In 20% (25 patients) of them, despite negative genetic testing, the clinical diagnosis had been considered definite.

The mean age of clinical hypercholesterolemia diagnosis was 29 ± 17 SD. However, there was a delay in genetic FH confirmation of more than 18 years. General clinical characteristics are shown in Table 2. A personal history of premature cardiovascular disease was very rare, whereas family history was present in nearly half of the cohort (41%). Moreover, most patients reported a family history of hypercholesteremia.

**Figure 2:**
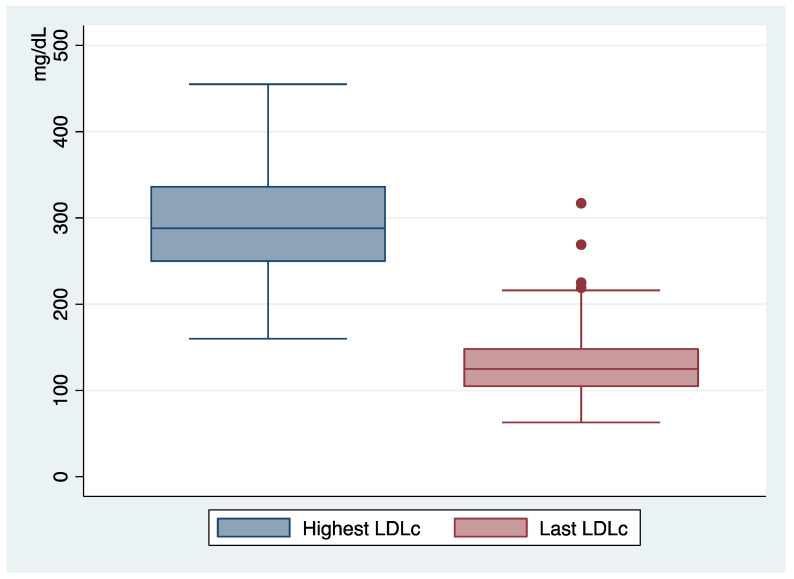
Highest LDLc and most recent LDLc recorded levels (md/dL).

When reviewing the medical history of this FH cohort, we found that their highest recorded LDLc levels were high, with a mean value of around 300 mg/dl (294 ± 65 SD). However, most patients were undergoing LLT and the last mean LDLc recorded levels were 133 ± 50 SD (Figure 2). These numbers should soon improve, as LLT was still being adjusted in those patients whose LDLc levels were off target. Moreover, one of the highest last LDLc levels was found in a pregnant woman whose LLT had to be interrupted.

Despite the relatively young mean age of the cohort (mean age 51 ± 19 SD), other important CV risk factors were found, with smoking being the most prevalent (22% considering smokers and previous smokers together. See Table 2). In 44% of FH patient participants, at least one additional modifiable cardiovascular risk factor (defined as DM, current or previous smoker, or HBP) was identified.

Women from this cohort were older than the men (57 ± 19 SD vs. 45.5 ± 17 SD, *p* = 0.02). Both dyslipidaemia diagnosis and genetic confirmation diagnosis were significantly established earlier in men than in women (25 years old ± 15 SD vs. 35 years old ± 19 SD, *p* = 0.02; and 43 ± 17 SD vs. 54 ± 19 SD, *p* = 0.02, respectively). Although both the higher LDLc recorded levels or the last ones were slightly worse in women, the difference was not statistically significant. Among other CV risk factors, women had significantly more HBP (*p* = 0.05). Nonetheless, there were no other significant differences found in cardiovascular risk factors, or personal or family history.

**Figure 3:**
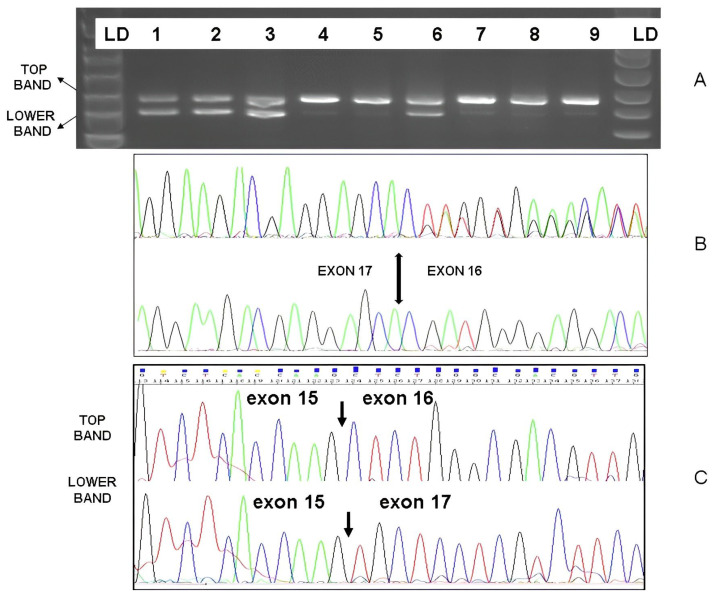
(**A**). Amplification of cDNA fragments from patients with (1,2,3,6) and without (4,5,7,8,9) and the *LDLR* intron 16 + 4 (c.2389+4 A>G) variant. The cDNA from leukocytes of heterozygous carriers showed two bands of the normal size (exon 16 present) and a shorter band that corresponded to the fragment lacking exon 16. (**B**). Sanger sequence (reverse strand) of PCR fragments from patients with (above sequence) and without (below sequence) the intron 16 variant. The amplification of the fragment without exon 16 resulted in two peaks with overlapping exon 16 and exon 15 sequences. (**C**) The top and lower bands from patients were excised from the agarose gels and sequenced, showing the normal exon 15–16 and alternative exon 15–17 sequences.

The LDLR intron 16 +4 (c.2389+4A>G) was one of the most common FH variants in our population, being the cause of FH in 19% of this cohort (11/58 patients). Patients without this intron variant amplified fragments of the normal size, while carriers of the intron 16 + 4G showed an additional shorter band (Figure 3). Sequencing of these PCR fragments confirmed the absence of exon 16 in these carriers. Moreover, this variant was absent in our control cohort of healthy patients with neither FH nor dyslipidaemia. In contrast to the global genetic FH cohort, most FH carriers of this variant were women (6/11). Nearly all of them had a family history of hypercholesterolemia (10/11) and half of them had a family history of premature ASCVD. Their LDLc highest recorded levels were significantly higher than the rest of the FH cohort (348 ± 61 SD vs. 282 ± 60 SD mg/dL, *p* = 0.002). Moreover, a female carrier presented tendon xanthoma and a male carrier presented both corneal arcus and tendon xanthomas.

One male patient was a homozygous carrier of the *LDLRAP1* c.207delC pathogenic variant. He was a 39-year-old male who had been diagnosed with dyslipidaemia at the age of 13, with 376 mg/dl being his highest LDLc recorded level. He also presented corneal arcus and tendon xanthomas, giving him the higher punctuation in the DLCN score from the cohort. However, he had no family history of dyslipidaemia nor premature ASCVD. Family screening confirmed that each parent was an asymptomatic carrier of one of the autosomal recessive pathogenic variants.

## 4. Discussion

In most patients, FH is caused by a single pathogenic variant in the three primary genes associated with heterozygous autosomal dominant FH [10]. Therefore, genetic testing for patients with suspected FH should, at a minimum, include the analysis of *LDLR*, *APOB*, and *PCSK9* [10]. More than 90% of reported FH-causing variants are in the genes encoding the low-density lipoprotein receptor (*LDLR*; OMIM #606945). In our cohort, as expected, most genetically confirmed FH patients had LP/P variants in this gene. *LDLR* LP/P pathogenic variants can be produced by numerous mechanisms including nonsense, missense, and a few synonymous variants; variants in the promoter and canonical splice sequences; and small insertions and deletions and large DNA rearrangements [23,24]. In our cohort, most pathogenic variants in *LDLR* were missense, followed by those which affect the splicing and nonsense variants. In this Spanish cohort, *LDLR* c.2389+4A>G was one of the single pathogenic variants that was the most prevalent, being present in nearly one in every five patients with genetically confirmed FH. Carriers of this variant had a high prevalence of familial history of premature ASCVD and hypercholesterolemia. In addition, interestingly, *LDLR* INT 16 + 4 A>G carriers had significantly worse LDLc levels than the rest of the genetically confirmed FH cohort. Moreover, to support its pathogenicity, we demonstrated that the change affected the splicing and that it was absent in a healthy control population of our own region (Figure 3).

Secondly, 5% to 10% of pathogenic variants are found in apolipoprotein B (*APOB*; OMIM #107730), and thirdly, less than 1% affect the proprotein convertase subtilisin–kexin type 9 gene (*PCSK9*; OMIM #607786) [10,14,25,26,27]. Multiple gain-of-function variants in *PCSK9* have been reported [28,29,30]. In our cohort, we did not find any pathogenic variant in this *PCSK9*. On the other hand, in the European population, the predominant pathogenic variant identified in FH cases affecting *APOB* is *APOB*p.Arg3527Gln (previously referred to as p.Arg3500Gln) [31]. Other *APOB* variants located outside of this region have been reported [32]. Nevertheless, its pathogenicity has been difficult to establish [33,34,35,36,37]. In this study, we identified two VUS affecting different regions of *APOB*. The carrier of *APOB* Asp1908Asn had another P variant (*LDLR* E288K) that was solely sufficient for an established genetic FH diagnosis. Nonetheless, in another patient (who had been diagnosed with hypercholesterolemia at 23 years old, whose highest LDLc recorded level was 232 mg/dL, and who had both hypercholesteremia and a premature ASCVD family history), genetic testing only identified the rare variant *APOB* Thr1558Ala. Although we believe that this variant may play a role in FH, it is classified as a variant of unknown significance.

In addition, in those patients with clinical FH suspicion but without LP/P variants in *LDLR*, *APOB*, and *PCSK9* genes, possible alternative molecular etiologies should be considered. For instance, it could be caused by pathogenic variants in the *APOE* gene, or in *LIPA* (lysosomal acid lipase deficiency autosomal recessive phenocopy) [10]. Moreover, there is an autosomal recessive form of hypercholesterolemia caused by biallelic pathogenic variants in the *LDLRAP1* gene, encoding the LDLR adaptor protein 1 (110). In the present study, to improve the genetic diagnosis yield, we evaluated all patients with an expanded panel including other genes: *LDLR*, *APOB*, *PCSK9*, *LDLRAP1*, *LIPA*, and *APOE*. As a result, we were able to identify one patient with autosomal recessive FH who was a homozygous carrier of the c.207delC *LDLRAP1* pathogenic variant. Remarkably, he was one of only two patients in this cohort who presented not only corneal arcus but also tendon xanthomas. Moreover, he not only had one of the highest LDLc levels recorded (376 mg/dl), but also had the highest DLCN score of the cohort. His parents were asymptomatic heterozygous carriers of the *LDLRAP1* variant. Thanks to the diagnosis of this rare autosomal recessive FH, accurate genetic counselling of recurrence risk information for relatives is now possible [10].

Despite FH being one of the most common genetic conditions and the main genetic disorder associated with premature ASCVD [6,13,14,15,20], FH is not only clinically underdiagnosed [16], but is also genetically underdiagnosed. For instance, in the CASCADE-FH registry, only 3% of all cases had genetic confirmation [38]. In this sense, in the Simon Broome FH register, only 13% of the total cohort had a DNA-confirmed diagnosis [39]. As in any other genetic condition, the yield of FH genetic testing depends on the pre-test probability. For instance, a pathogenic variant in one of the main three FH genes can be found in up to 60–80% of patients with a clinical “definite” FH diagnosis. However, the genetic yield is lower (21% to 44%) in those with a clinical “possible” diagnosis [26,40,41,42]. Likewise, in our cohort, the global genetic yield for genetic FH was 32%. Nonetheless, if only those patients with probable to definite clinical FH diagnosis were sequenced, this genetic yield would improve to 42%.

Although the diagnosis of FH could be made based on clinical findings alone, genetic testing is of the utmost importance [10]. It has been reported that the risk for ASCVD was higher in FH pathogenic variant carriers compared with non-carriers, at any LDLc value [10,43]. Even small mean reductions in LDLc levels with LLT were significantly associated with delayed CV events and prolonged survival of FH patients [44]. Moreover, patients with FH diagnosed through DNA testing had a higher perceived efficacy of medication (79). Thus, genetic results provide not only prognostic information and the ability to perform personalised risk stratification [10], but also encourage intensive LLT both for patients and physicians [10].

Most untreated FH patients can experience a CV event or death by the second decade of life [44]. Their risk for coronary heart disease is over 50% for men by the age of 50 and at least 30% in women aged 60 years old [45,46]. However, if FH patients are treated with LLT from an early age, their ASCVD risk could be substantially reduced [4]. What is more, when FH is properly treated from an early age, the risk of myocardial infarction above 55 years old could be reduced to that of the general population [4]. However, in general, FH is not only an underdiagnosed but also an undertreated condition. According to the CASCADE-FH registry [38], only 45% of patients were under statin treatment. In this sense, there was a high prevalence of family history of premature ASCVD in patients with FH genetic diagnosis from our cohort (41%). However, we found an encouraging low prevalence of personal history of premature ASCVD (5%). We believe that this low rate of CV events may be due to the early beginning of LLT in the population and high rates of LLT (95%, considering one untreated woman due to pregnancy). In our cohort, the genetic confirmation of FH was achieved at a mean age of 48 years old, similar to that in the national CASCADE-FH registry [38]. However, most patients had already been diagnosed with hypercholesterolemia and subsequently began LLT before the age of 30. Moreover, 79% of our patients received >1 LLT medication. This percentage, although still improvable, is better than those reported in other registries, such as 45% in the CASCADE-FH registry [38]. Moreover, thanks to the availability of IPCSK9 (proprotein convertase subtilisin/kexin type 9 inhibitors), better reductions in LDLc levels are expected.

In addition, ASCVD risk reductions were found both in men and women in most studies [4]. However, other studies about LLT in FH patients showed excess mortality in women [39,47] and suggested that women with FH may have not been properly treated [47,48,49]. In this sense, it has been reported that LLT is initiated about 5 years earlier in men than in women [39]. Unfortunately, LLT prescribing rates have been shown to be lower in women than in men in the general population [50]. Women’s ASCVD risk can be underestimated due to the misperception that they are ‘protected’ before the menopause [51]. In general, women are less likely to be prescribed according to evidence-based guidelines, and less aggressively treated in cardiology care for both primary and secondary prevention [39,52]. In addition, statins are not recommended during pregnancy and breastfeeding. In the present study, we did not find statistically significant differences between genders for LDLc highest and last control levels. However, dyslipidaemia diagnosis and genetic confirmation were indeed established significantly earlier in men. Moreover, there was a pregnant woman with high LDLc levels, whose LLT had been interrupted due to pregnancy.

On the other hand, our study reaffirms the importance of identifying other traditional modifiable cardiovascular risk factors in patients with FH. At least one additional modifiable cardiovascular risk factor was identified in 44% of the already high-risk FH patients (Table 1). These findings are largely consistent with previous studies and reinforce the importance of comprehensive preventive care to minimise cardiovascular risk in those with FH [38].

Apart from this, genetic testing also enables cascade testing [10], a major opportunity to identify relatives at high ASCVD risk, with a grade I recommendation, supported by extensive epidemiological and cost analyses data [10]. For instance, in this cohort, thanks to genetic results, the first-degree relatives of all FH patients can be genetically tested for the identified LP/P variant. If any relative is a carrier of the LP/P variant, clinical follow-ups to control LDLc levels with LLT from a young age can be scheduled, and so on with their first-degree relatives. In summary, prevention strategies can be extended to all available relatives with concealed high cardiovascular risk.

Increasing public and health professional awareness about FH is essential [53,54,55]. Current evidence suggests that early detection of FH and cascade testing meet most of the criteria for a worthwhile screening program [55]. Primary care is a key target area to increase identification of new index cases. For instance, child–parent screening was feasible in primary care practices at routine child immunisation visits [56]. Moreover, coronary care units are other settings where FH may be identified [20,55]. An interesting meta-analysis by Beheshti et al. reported an FH prevalence that was 20-fold higher among patients with premature ischemic heart disease, and 23-fold higher among those with hypercholesterolemia with LDL levels ≥ 190 mg/dl [57]. As a result, we believe that opportunistic genetic screening in high-risk populations, including patients with high LDLc levels or presenting premature ASCVD, could be cost-effective.

## 5. Limitations

Gene dosage for the identification of large deletions was not routinely performed in our patients. Deletions of multiple exons had been identified in some patients with high LDLc values and highly penetrant familial FH, especially in the *LDLR* gene. Thus, our study could have underestimated the rate of pathogenic variant carriers by not including these rare forms of FH. If routinely performed, the genetic yield could have been even higher.

## 6. Conclusions

In this Spanish cohort, genetic testing identified FH in nearly one in every three patients with clinical FH suspicion. Genetic FH diagnosis was established in their fourth decade of life and significantly later for women. The *LDLR* c.2389+4A>G was one of the most common FH variant and its carriers had significantly worse LDLc highest recorded levels. An autosomal recessive FH patient (homozygous carrier of the *LDLRAP1* c.207delC pathogenic variant), was also found.

Although the presence of a family history of premature ASCVD and other CV risk factors was frequent (41% and 44%), nearly 95% of them were on statins and their personal history premature ASCVD was extremely low. Our finding reaffirms the importance of primary prevention strategies from a young age in all FH patients, with particular attention paid to women with FH.

## Figures and Tables

**Table 1:** Genetic variants identified via NGS which may play a role in FH.

Gene	Transcript	Protein	cDNA	gnomAD v2.1 (European Non-Finnish)	Carriers	ACMG
*LDLR*	NM_000527	(p.Glu31Ter)	c.91G>T	-	1	P
*LDLR*	NM_000527	(p.Cys155Tyr)	c.464G>A	-	1	P
*LDLR*	NM_000527	(p.Glu174Ter)	c.520G>T	-	1	P
*LDLR*	NM_000527	(p.Ser177Leu)	c.530C>T	-	1	P
*LDLR*	NM_000527	(p.Glu288Lys)	c.862G>A	-	1	P
*LDLR*	NM_000527	(p.Asp354Asn)	c.1060G>A	-	1	LP
*LDLR*	NM_000527	(p.Arg406Trp)	c.1216C>T	-	1	P
*LDLR*	NM_000527	(p.Arg416Trp)	c.1246C>T	1/113,496	3	P
*LDLR*	NM_000527	(p.Val429Met)	c.1285G>A	1/113,592	11	P
*LDLR*	NM_000527	(p.Trp490Ter)	c.1470G>A	-	1	P
*LDLR*	NM_000527	(p.Phe530SerfsTer20)	c.1589_1614del		2	P
*LDLR*	NM_000527	(p.Gly592Glu)	c.1775G>A	15/129,176	7	P
*LDLR*	NM_000527	(p.Leu599Ser)	c.1796T>C	-	1	LP
*LDLR*	NM_000527	(p.Glu602Ter)	c.1804G>T	-	1	P
*LDLR*	NM_000527	(p.Phe655Leu)	c.1965C>G	-	1	LP
*LDLR*	NM_000527	(p.Leu658Pro)	c.1973T>C	-	2	LP
*LDLR*	NM_000527	(p.Asp700Gly)	c.2099A>G	-	1	LP
*LDLR*	NM_000527	(p.Leu799PhefsTer127)	c.2395_2404del		1	P
*LDLR*	NM_000527	(p.Arg814Gln)	c.2441G>A	-	2	LP
*LDLR*	NM_000527	Splicing	c.313+2insT	-	2	P
*LDLR*	NM_000527	Splicing	c.1987+1G>A	-	1	P
*LDLR*	NM_000527	Splicing	c.1988-2A>T	-	1	P
*LDLR*	NM_000527	Splicing	c.2389+4A>G	1/113,644	11	P
*LDLR*	NM_000527	Copy number variant	c.2390-2583del	-	1	P
*LDLRAP1*	NM_015627	p.Ala70ProfsTer19	c.207delC	-	1	P
*APOB*	NM_000384	(p.Thr1558Ala)	c.4672A>G	-	1	VUS
*APOB*	NM_000384	(p.Asp1908Asn)	c.5722G>A	2/129,088	1	VUS
*LDLR*	NM_000527	(p.Asn297His)	c.889A>C	-	1	VUS
*LDLR*	NM_000527	(p.Ala606Ser)	c.1816G>T	30/129,154	1	VUS
*LDLR*	NM_000527	(p.Hys656Asn)	c.1966C>A	3/113,732	1	VUS
*LDLR*	NM_000527	(p.Arg253Gln)	c.758G>A	-	1	VUS

FH: familial hypercholesterolemia; LP: likely pathogenic; P: pathogenic; VUS: variant of unknown significance.

**Table 2:** Clinical characteristics of patients with genetic diagnosis of FH.

Identified Patients (58)	N/Mean	Frequency/SD
Men	31	53.5%
Women	27	46.5%
Current age (years), mean ± SD	51	±19 SD
Age at dyslipidaemia diagnosis	29	±17 SD
Age at definite genetic diagnosis	48	±19 SD
Other cardiovascular risk factors		
Previous smoker/ Current smoker	13	22%
High BP	11	19%
DM	8	14%
At least 1 cardiovascular risk factor	26	45%
Kidney failure	1	2%
Peripheral vascular disease	1	2%
Personal history of PCVD	3	5%
Family history of PCVD	24	41%
Family history of hypercholesteremia	53	91%
Corneal arcus and/or tendon xanthomas	9	15.5%
Lipid profile		
Highest LDLc level (mg/dL)	294	±65 SD
Last LDLc level (mg/dL)	133	±50 SD
LpA (nmol/L)	34	±74 SD
Medical treatment		
None	3	5%
Statins	9	15.5%
Statins + ezetimibe	39	67%
Statins + ezetimibe + IPCSK9	7	12%
DLCN criteria		
<3	6	10%
3–5 possible	8	14%
6–8 probable	21	36%
>8 definite	23	40%

FH: familial hypercholesterolemia; SD: standard derivation; BP: blood pressure; DM: diabetes mellitus; LDLc: low-density lipoprotein cholesterol; PCVD: premature cardiovascular disease; DLCN: Dutch Clinical Lipid Network; IPCSK9: proprotein convertase subtilisin/kexin type 9 inhibitors.

## Data Availability

Not applicable.
